# Perceptions on the current content and pedagogical approaches used in end-of-life care education among undergraduate nursing students: a qualitative, descriptive study

**DOI:** 10.1186/s12909-022-03625-y

**Published:** 2022-07-16

**Authors:** Wenjing Cao, Chunyan Li, Qianqian Zhang, Huiru Tong

**Affiliations:** 1 Nursing School, Xiangnan University, Chenzhou, Hunan China; 2 Nursing School, Hunan University of Chinese Medicine, Changsha, Hunan China; 3 Foreign Language Department, Guangxi University of Chinese Medicine, Nanning, Guangxi China

**Keywords:** End-of-life care, Undergraduate education, Qualitative research

## Abstract

**Background:**

With the aging of the population, high rates of cancer and comorbidity complexity, the end-of-life care for patients will be ever more important. Nurses have always played an essential role in end-of-life care. Insufficient education and training in end-of-life care has been regarded as a major reason of inadequate symptom recognition, symptom management, and communication which results in mental trauma for both the patient’s family and attending health care providers. Undergraduate nurses do end-of-life care as part of their clinical learning. However, undergraduate nurses’ perceptions of the education they received about end-of-life care are not documented.

**Objective:**

This study aimed to critically explore the current state of education regarding end-of-life care from the perspectives of undergraduate nurses.

**Methods:**

We used a descriptive qualitative design. Face-to-face semi-structured interviews were conducted from May to August 2020, with a purposive sample of 15 fourth-year undergraduate nurses who finished the internship. Data were transcribed verbatim and analyzed using content analysis.

**Findings:**

Three main themes relating to undergraduate nurses’ experiences of end-of-life care education emerged from the thematic analysis: 1) Universities provide foundational knowledge about end-of-life care, but it still needs improvement; 2) Clinical practice consolidates and drives undergraduate nurses’ knowledge, skills and confidence about end-of-life care; and 3) cultural attitudes of patients’ family toward disease and death sometimes impedes learning and knowledge translation about end-of-life care.

**Conclusion:**

Undergraduate nursing students benefit from not only theoretical content delivered in the university setting but also practice happened on clinical placement. The current undergraduate curriculum, related to end-of-life care, is disjointed. Meanwhile, undergraduate nurses’ learning and knowledge translation of end-of-life care are impeded by cultural attitudes toward disease and death.

## Introduction

Along with medicine advancement, the emphasis of care has been traditionally centered on curative measures and restoring health to a preillness state. However, increasing patient age, high rates of cancer and comorbidity complexity have led to the fact that health care providers should also focus on those individuals at the end-of-life when death is unavoidable. One study identified that in 2017, there were 24.5 million incident cancer cases worldwide (16.8 million without nonmelanoma skin cancer [NMSC]) and 9.6 million people died from cancer [1]. In China, there are approximately 7.5 million deaths each year, among which 80% (6 million people) call for end-of-life care, which emphasizes symptom control and meeting the complex care needs of patients with life-limiting conditions and their families to optimize quality-of-life [2].

End-of-life care refers to the medical care and support provided in the time surrounding death [3]. The end-of-life experience is unique and personal for each individual where most agree that they wish to avoid suffering during this time and others wish to prolong life at all costs. End-of-life care may be undertaken by doctors, physicians, nurses, paramedics or volunteer. However, nurses are primarily responsible for it [4]. One study showed that nurses are moral agents who are deeply invested in the moral integrity of end-of-life care involving assisted death [5]. Furthermore, another study also indicated that nurses have always played a critical role in caring for the end-of-life patients [6]. Nurses are expected to provide the highest quality of care for dying patients and their families. Patients at the end-of-life stage have reported experiencing several distressing symptoms such as unsatisfied thirst, dyspnea, chronic pain, anxiety, depression, and difficulty sleeping, as well as delirium and agitation during the final stages of their illness trajectory [7, 8]. It is imperative that health care providers understand these signs and symptoms of patient discomfort to help support patients through the dying process as safe, dignified, and comfortable as medically possible [9].

Nurses have identified insufficient knowledge as one of the largest barriers to providing care for the end-of-life population [10]. Insufficient education and training in end-of-life care has been regarded as a major factor to inadequate symptom recognition, symptom management, and communication [11]. Given that end-of-life care is an important nursing responsibility, this phenomenon is not only surprising but also concerning. Previous study showed that physical and psychological side effects of end-of-life patients frequently go unrecognized by health care providers and thus remain untreated [12]. This situation results in mental trauma for both the patients’family and attending health care providers [13–15]. Studies have shown that end-of-life patient management often cause emotional exhaustion and professional burnout for health care providers [16]. To make things worse, unappropriate end-of-life care are associated with significant adverse events which include increased the extraordinary costs of burdensome, increased use of nonbeneficial treatments, and increased conflict over goals of care [17, 18]. Thus, national healthcare initiatives have realized that the importance of end-of-life care cannot be underestimated and put more focus on the provision of appropriate, high-quality end-of-life care.

Educational gap has been recognized as a barrier to evidence-based practice [19]. A Japanese study found that nurses needed opportunities to get access to education about end-of-life patients psychological care of the family and health care professionals, ethical decisions concerning treatment withdrawal, and communication with the family [20]. Considering the majority of undergraduate nurses practice end-of-life care as part of their clinical learning experiences, it is essential to prepare students for ethical practice standards. In other words, undergraduate nursing students need the most education in improving knowledge and skill of end-of-life care and management of end-of-life patients.

Some studies have shown that educational institutions have incorporated end-of-life care into the undergraduate nursing education and explored the end-of-life nursing education consortium curriculum as core curriculum and initiated faculty training [21]. And only 25% of nursing schools across the United States have emphasized end-of-life care in nurse education [21].

Whilst it is encouraging to see researchers explore nurses’ knowledge and experience about end-of-life care, much less is known about undergraduate nursing students’ level of training, knowledge, and skills on end-of-life care. To our knowledge, few studies available assessing what is taught in undergraduate nursing programs about end-of-life care, nor is there literature exploring how end-of-life care content in undergraduate nursing curricula was undertaken. Therefore, in this exploratory, descriptive study, we aimed to understand the depth, breadth and content of undergraduate nursing education around end-of-life care in order to advance future teaching and learning programs in this area.

Specifically, the following research questions were sought to answer in this study:

- Question 1: What are undergraduate nursing students’ perceptions of the current content and pedagogical approaches used in end-of-life care education?
- Question 2: What are the aspects of end-of-life care related clinical practice for which undergraduate nursing students perceive they are well or poorly prepared?

## Methods

This study used a qualitative, descriptive design [22]. The approach enabled flexibility in data collection and analysis to obtain rich information, providing a comprehensive summary to the broad insights among the study participants [23]. Ethical approval was received from XiangNan University.

### Participant recruitment and selection

Participants were selected from regional universities in Hunan Province, China. First, we selected three from the regional universities by random number in Hunan Province list. Then a purposive sampling technique was used to recruit potential participants to produce a representative sample [24]. The first author contacted the head of the nursing school in each study university to initiate this possible collaboration, recruiting students, and obtaining approval for the study. The head of the nursing school in each study university was very supportive of the study, and they helped us recruit students by distributing email advertisements as well as flyer in classroom to fourth-year undergraduate nursing student who finished the internship. Interested students contacted primary author and, a verbal explanation of the project, an information sheet and consent form were sent to each respondent. Participation was then confirmed by written informed consent. The process of recruitment and data collection ended when the saturation of data was achieved [25]. Data saturation was described as being achieved when no new categories emerge from the data [26].

### Data collection

Data were collected from fifteen participants through semi-structured interviews between May to August 2020, digitally recorded and transcribed verbatim. Confidentiality and anonymity were ensured, and a semi-structured interview guide was used to elicit information about participants’ experiences of end-of-life care education [7]. An interview guide was developed based on the literature [6, 27] and the clinical expertise of the authors. To eliminate disruptions, the interviews were conducted in the setting preferred by each participant and were arranged at a date and time convenient for the participant. Each participant conducted a face-to-face interview. The interviewers were two female researchers with a master or doctoral degree in nursing. Both interviewers had received extensive training on qualitative methodology, research ethics, general skills of conducting in-depth interviews and have interest in end-of-life education. The interviewers were not in close contact (e.g. friendships) with participants. Students were asked to describe their experiences of end-of-life care education, questions included; ‘Can you describe the experiences of end-of-life care education when you learned it in university?’, ‘What has helped you in the learning of end-of-life care?’, ‘What has negatively affected your experience of learning knowledge, skills about end-of-life care? ‘and ‘Any suggestions for improvements in relation to your learning and knowledge translation about end-of-life care? Participants were encouraged to share as much of their experience as possible. Interviews were recorded and lasted between 25- and 44-min. Field notes were made during the interviews. Interviews were recorded. All the participants finished their interview. No one else was present besides the participants and researchers during the interview. Transcripts were managed using the NVivo 11 software (QSR International, Melbourne, VIC).

### Data analysis

Qualitative content analysis was used as the methodological approach and we applied the COREQ (consolidated criteria for re-porting qualitative research) checklist to report our findings. Content analysis was used to underpin the study. Based on this method, we classified relevant ideas deductively and inductively in a systematic process. We built a coding frame through creating subcategories to categories of higher order by assigning text messages. The categories and the coding frame were discussed and validated by all the researchers. Themes were derived from the data.

Sample audit trai for analysis was illustrated in Table 1. Classification of text segments was undertaken separately by Chun yan Li and Qianqian Zhang to approximate reliability through intersubjective consensus. We recruited interview participants until thematic saturation was reached.

**Table 1:** Sample audit trail for analysis

Text segment		Findings	Category	Theme
“…learn something actually.”		Have learn something from university	Students learn something about end of life care from school	university provides foundational knowledge about end-of-life care, but it still needs be improved
“…what we leant was just several lectures in class period, it cannot be remembered.”		Learn is not enough	Knowledge is not enough so students can not consolidate knowledge well	
“…Practice makes me understand what I learn in school.”		clinical practice help students understand knowledge and skills	clinical practice is very useful in understanding knowledge	clinical practice drives and consolidates knowledge, skills and confidence about end-of-life care
“…clinical practice let me learn much more and feel confidence to do end-of -life care.”		Clinical practice improves confidence	clinical practice generates confidence in care	
“…In China, patients’ family decline to talk death. We can not do end-of -life care as we planned.”		cultural attitudes toward death sometimes make end-of -life care can not be scheduled	cultural attitudes of patients’ family toward death affect practice	cultural attitudes of patients’ family toward disease and death sometimes impedes learning and knowledge translation about end-of-life care
“…family members have inaccurate optimism about the diseases and hard to accept end-of-life care.		cultural attitudes toward disease make nurse students beconsumed with carrying out lifesaving measures	cultural attitudes of patients’ family toward disease affect practice	

### Rigour

Different strategies were adopted aimed at ensuring dependability, transferability, confirmability and credibility throughout the research process: 1) We developed the interview guide based on the literature [28]; 2) Before data collection, two pilot interviews were conducted with three nursing students to test whether the format was clear and comprehensible and no changes were needed; 3) two researchers with different clinical and research lenses performed the entire process independently; at each step, they shared and agreed upon the findings ensuring veracity of the theme identification [29]; and 4) at the end, the consistency of the analysis was ensured by the entire research team which enhanced inter-rater reliability and triangulation during the data analysis. We also returned our finding to the participants.

## Findings

A total of 15 students (13 female, 2 male), aging from 20 to 24 year, finished the interviews. More than 70% comes from towns and are the only child in their family, with interning time ranging from 40 weeks to 44 weeks.

The coding tree identified three categories of higher order aligned to our research question:

1) Universities provide foundational knowledge about end-of-life care, but it still needs improvement; 2) clinical practice consolidates and drives undergraduate nurses’ knowledge, skills and confidence about end-of-life care; and 3) cultural attitudes of patient’s family toward disease and death sometimes impedes learning and knowledge translation about end-of-life care.

### Theme one: university provides foundational knowledge about end-of-life care, but it still needs improvement

Participants identified that the end-of-life care education they received at university was often limited and lacking detail, as one participant vulnerably shared:

Yes, I think we did get taught things at university. In fact, I can list the things that’s meant to be considered, but then we didn’t really delve into it. They just said, “Recognition of advancing disease states, continuity of care, and decision making are very important in end-of-life care. But it is not easy.” And then how to recognize advancing disease states? What is legal considerations for management, and ethical decision making in EOL patients?

(P2)

Some participant illustrates that end-of-life care education provided by the university was brief and disjointed, as seen in the quote here in after:

Honestly speaking, end-of-life care education provided by the university was very brief and it was just several lectures in class period, it has slipped my memory. I feel like the buddy nurses reiterated everything when I went back on placements.

(P9)

Insufficient time as well as the complexities of caring, was acknowledged by participants as important factors for the provision of foundational knowledge rather than more comprehensive, in-depth teaching regarding end-of-life care.

End-of-life care is only a small part of the required curriculum, very little time is spent on this content. I know we’re only looking at this aspect, but there’s a lot of things that you have to do about end-of-life care, which consisted of pain assessment of verbal or nonverbal patients, ethical decision-making methods, symptom recognition and management.

(P8)

Most undergraduate nursing students had difficulty in recalling what they had been taught about end-of-life care, when they had been taught about end-of-life care, and where the end-of-life care education occurred. However, participants were aware that the education they received about end-of-life care was necessary and relevant despite the fact that it was basic.

They did do it. They definitely stepped us through how to do end-of-life care, including predeath care (from diagnosis and discussion of ethical problems to the decision on treatment), care during active dying (from withdrawal of life support to death), and after-death care (bereavement care). But I just don’t have any memory of it. And I have to admit that the knowledge we received was necessary and relevant.

(P5)

### Theme two: clinical practice drives and consolidates knowledge, skills and confidence about end-of-life care

Through the interviews, it was evident that clinical practice drives and consolidates knowledge, skills and confidence about end-of-life care. This is clearly illustrated in narratives below, in which a participant outlines the clinical environment was where they were able to consolidate their theoretical knowledge and apply this knowledge in practice.:

Authentic learning occurred in the practice setting. My buddy RN (RN responsible for supervising student during educational clinical placement) would bring us to the bedside of a dying patient and then she’d watch us, and prompt us to do symptom recognition and management. And if she noticed we were a bit unsure she’d remind us what we should be doing with the end-of-life care. And always reminding us to pay attention to the humane care of the dying patients.

(P3)

Participants identified that clinical practice is needed to enhance the application of theory related to end-of-life care. Only by exposure to the clinical setting can the knowledge and skills related to end-of-life care become practical. The importance of the RN buddy, to facilitate students to develop and consolidate knowledge, skills, was also identified by participants:

It’s no exaggeration to say exposure to the clinical setting really made a big difference. The nursing buddies are who helped me the most to translate theory to practice. On clinical, the nurse buddy would tell us how to do end-of-life care and they would usually step us through on how we should manage the pain assessment or other symptoms. Sometimes we’re watching them and they’re showing us what we should be doing with them.

(P7)

### Theme three: cultural attitudes of patient’s family toward disease and death sometimes impedes learning and knowledge translation about end-of-life care

Participants highlighted cultural context adversely affecting learning and knowledge translation about end-of-life care. They described that denial of Death caused confusion and impacted their ability to translate best practice:

We live in a death-denying society and with the technology advances, hospitals confront system-wide challenges to care of the dying. Expectations for medical therapies, and especially life-prolonging treatments, are inflated. For example, when we wanted to conduct the withdrawal of life-saving treatment, family members of end-of-life patients did not prepare for the death of end-of-life patients. It’s confusing. In this cultural context, it is enormously, perhaps impossibly, difficult to apply what I have learned about end-of-life care.

(P1)

Understanding of end-of-life care is a critical and complex subject. Our patients seem to expect hospitals to offer the most sophisticated strategies to avoid death’s inevitable approach. Meanwhile, in the context of end-of-life care, there are so many things we have to do. However, sometimes we lacked opportunities to participate on end-of-life care. A major challenge to offering end-of-life care clinical experiences is cultural attitudes toward disease and death.

(P4)

Influenced by traditional Chinese culture, people in China think it is unlucky to talk about death, and it may bring bad luck to death prematurely. It seems that talking about death is a taboo in China. In hospital, we should be sensitive toward the cultural influence during advance care planning and respect family-oriented decision-making. Sometimes, I missed out on learning opportunities because of this situation.

(P13)

## Discussion

This descriptive qualitative study explored undergraduate nurses’ perceptions about the current content and pedagogical approaches used in end-of-life care education. We identified three themes:1) Universities provide foundational knowledge about end-of-life care, but it still need improved; 2) Clinical practice consolidates and drives undergraduate nurses’ knowledge, skills and confidence about tend-of-life care; and 3) cultural attitudes of patients’ family toward disease and death sometimes impedes learning and knowledge translation about end-of-life care.

Nurses play a crucial, complementary role in improving the quality of end-of-life care through adequate communication, symptom recognition, and symptom management in end-of-life population, but for nursing students, they have to adequately prepared for this role given that nursing students will be the main healthcare workforce in the future [30].

Price et al. [31] found that end-of-life care education in university may not only good for undergraduate nurses’ professional development, but also good for the palliative care patients. Undergraduate nursing education in improving nursing perceptions of end-of-life patient management, decreasing levels of nursing stress, and improving interdisciplinary collaboration and communication at the end-of-life was stressed all the time [32, 33]. Though end-of-life care, students do not only learn about symptom relief, but they interact with interdisciplinary teams, where they are able to find role model to learn how to deal with complex psychological, social, ethical, cultural, and religious situations [34, 35]. Education experts state that there is a strong contribution of end-of-life care education to professionalism [35].

Though there is a wealth of research exploring ongoing end-of-life care education undergraduate nurses’ personal perceptions of their education about end-of-life care are not well documented. In addition, recently studies had also shown that undergraduate education in end-of-life care area is considered inadequate [36, 37]. Furthermore, Li, Jing et al. indicated that end-of-life care education is desperately needing an improved dissemination plan within low-and middle-income countries where education on end-of-life care is lagging [38].

Our study showed that undergraduate student nurses perceived the end-of-life care education they received at university was so superficial that it was easy to be forgotten, and they felt without the instruction and learning support from their clinical teacher, RN buddy or preceptor, they could not manage end-of-life care in clinical practice. This result is consistent with the previous study that reported students, despite expressing positive attitudes toward patient-centeredness teaching models, feel that they do not receive sufficient end-of-life training during their medical studies to attain the level of competent behavior needed in today’s challenging hospital environment [39]. One descriptive correlational study conducted in China found that third year of nursing students had low level of knowledge regarding end-of-life care and there was a need for integrating end-of-life care education into nursing curriculum in China [40]. In addition, a qualitative study exploring nursing students’ perspectives on and attitudes towards hospice care in China repotted those medical educational institutions and the government should take action to increase end-of -life care training for nursing students [41].

The need for more education on end-of-life care has implications for curriculum development in undergraduate nursing programmes, Maria Dimoula et al. suggested that structured courses in end-of-life care might be a core part of undergraduate nursing education [42].

In light of this, given the importance of end-of –life care and the limit learning experience of nursing students refer to end-of –life care, we suggest that nurse students need more training relating to end-of-life care. Our finding may therefore highlight there is potential for improvement and innovation in relation to how undergraduate nurses are educated about systematic and focused end-of-life care [43].

One noticeable result of this study is that clinical practice consolidates learning and promotes knowledge translation about end-of-life care. Supportive and facilitative clinical teacher, mentor or preceptor was shown to have significant influence on developing nursing students’ clinical knowledge and skills [44]. It has been identified that knowledge translation is a collaborative endeavour and collaborative and collegial relationships between education providers and clinical partners are imperative. Papastavrou et al. [45] revealed that the clinical learning environment and supervision should be a significant element of the development of future nurses’ clinical competence. We found although the education about end-of-life care at university was often basic and foundational, participants in this study identified that clinical practice was a major influence on their clinical learning. Results from our current study offer an interesting insight into the alignment of theory to practice. This result is agreed with the previous study that reported providing theoretical knowledge that can then be applied in the clinical setting was of great importance [46]. This finding has also been identified by Jafari M [47] who revealed that it was when students have the opportunity for clinical practice associated with the didactic content that they learn best. According to this finding, there is a pressing need to implement knowledge translation strategies that enable consolidation of complex theoretical constructs into the clinical arena.

Cultural attitudes toward disease and death impedes learning and knowledge translation about end-of-life care were the third finding that emerged from analysis of the interview data. Participants in this study cited that death-denying attitudes made it difficult to facilitate knowledge translation. We live in a death-denying society and one that becomes only more so as technology advances. In this cultural context, expectations for medical therapies, and especially life-prolonging treatments, are inflated [48]. Without acquiescence that life comes to an end, it is enormously, perhaps impossibly, difficult to provide good end-of-life care. One study concluded that nursing students do not have a negative attitude toward caring for dying patients [6]. Some studies demonstrates that the didactic and clinical experiences of end-of-life care in nursing programs were inadequate due to the denial of death [49, 50] However, previous study showed that death education can change both students’ and patients’ attitudes toward death [51]. Evidence suggested that death education should be strengthened in China [52]. It is important for nurses to be sensitive to the cultural influence and encourage the preparation of patients and families who are unprepared for death. Such a situation is a very good opportunity for students to learn about end-of-life care. Balante J et al. thinks stakeholders, such as the nursing workforce, need to play an active role in providing a culturally inclusive workplace to encourage students to experience end-of-life care [53].

This study also has several potential limitations which are worth considering. Firstly, participants were interviewed retrospectively about their experiences and perceptions in relation to their education about end-of-life care, some degree of recall bias could not be ruled out. Secondly, we also only interviewed fourth-year undergraduate nursing student from regional universities in Hunan Province, China, which limits its generalizability to the broader undergraduate nursing student in China. However, as fourth-year students are in the final stages of preparation for graduate practice, we felt it was of great importance to pay attention to this cohort.

## Conclusion

End-of-life care is an important nursing competency and has been incorporated into the baccalaureate nursing curriculum in some countries. Yet undergraduate nurses’ perceptions of the education they receive about end-of-life care has not been explored and are poorly understood. Our study suggests that undergraduate nursing students benefit from theoretical content of end-of-life care delivered in the university setting. On the other hand, we importantly demonstrate the alignment of theory to practice during undergraduate nurses’ clinical placement. In this study, we also identified that despite the call for inclusion of end-of-life care in the undergraduate curriculum, death-denying attitudes impedes knowledge translation related to end-of-life care.

## Data Availability

The datasets used and analysed during the current study are available from the corresponding author upon a reasonable request.
